# Effects of telerehabilitation-based physical therapy for individuals with Parkinson’s disease: A systematic review protocol

**DOI:** 10.1371/journal.pone.0342771

**Published:** 2026-03-06

**Authors:** Liliane Santos de Vasconcellos, Raíssa Souza Taveira, Luciana Protásio de Melo, Lorenna Raquel Dantas de Macêdo Borges, Lorenna Marques de Melo Santiago, Larissa Coutinho de Lucena, Tatiana Souza Ribeiro

**Affiliations:** 1 Federal University of Rio Grande do Norte, Natal, Brazil; 2 Santos Dumont Institute, Brazil,; 3 State University of Paraíba, Brazil; University of South Australia, AUSTRALIA

## Abstract

**Introduction:**

The use of telerehabilitation (TR) may increase in the next years due to the appreciation and acceptance of individuals with Parkinson’s Disease (PD). The objective of the present study is to evaluate the effects of physical therapy interventions administered remotely (TR) in individuals with PD.

**Methods and analysis:**

This is a systematic review protocol in which randomized and quasi-randomized clinical trials of studies involving adults (≥ 18 years old) diagnosed with PD undergoing TR will be included. It is expected to provide evidence of the effects of TR in individuals with PD in terms of motor function; gait; balance; quality of life; number of falls; adverse effects; individual adherence to TR and fear of falling. Data will be synthesized using the Review Manager software.

**Registration details:**

This systematic review protocol was registered on the Open Science Framework (https://doi.org/10.17605/OSF.IO/ZVPX5-https://osf.io/zvpx5).

## Introduction

Parkinson’s disease (PD) is one of the fastest-growing neurological disorders worldwide and represents a major public health challenge, affecting approximately 1% of the population aged 65 and older [1–3]. It is characterized by motor (e.g., bradykinesia, stiffness, postural instability, resting tremor, and episodes of freezing of gait) [4,5] and non-motor symptoms (e.g., constipation, hypotension, and depression) and affects mobility, social interaction, and mental health [6]. The physical therapist is the professional in the multidisciplinary team aims to improve functional capacity and minimize secondary complications through rehabilitation, education, and support [7].

In this context, telerehabilitation – TR (i.e., image-based, sensor-based, or virtual reality) uses information and communication technologies to support the continuity of care, reduce costs, and provide greater accessibility to the services [8]. The barrier of the traditional rehabilitation concept that requires touching the individual during treatment has been surpassed by sophisticated optics systems and technologies that provide remote evaluation and intervention [9].

A systematic review compiled studies involving 263 individuals with Parkinson’s disease and found a positive effect of short-term virtual reality (VR) exercise on step and stride length. VR-based interventions and conventional physiotherapy may have similar effects on gait, balance, and quality of life [4]. Telehealth grew during the coronavirus disease pandemic and incorporated a wide range of technologies and digital strategies to improve the response of health systems [10]. This approach has already been used in various health specialties, including neurology. According to Chen et al., more than 85% of neurology departments in the USA use or will use telehealth strategies in clinical practice [11]. Recent studies also demonstrated that individuals were satisfied with telehealth applications and will continue after the pandemic [12].

A recent review demonstrated the efficacy of TR and monitoring to control tiredness, pain, and depression and promote physical activity and pain management in individuals with neurological conditions [13]. The increasing interest in TR suggests that this area will continue to expand [14,15] and be implemented in low- and middle-income countries [16].

TR allows the evaluation and treatment of individuals and dialogue with professionals through videoconferences [11]. In this context, TR may help individuals with PD who experience barriers that limit their access to face-to-face therapeutic assistance (e.g., distance, locomotion difficulties, financial burden, and lack of time) [17].

The literature demonstrates that TR reduced speed of progression of motor symptoms in PD, ensuring a viable and beneficial alternative. Also, individuals with PD prefer TR to face-to-face therapeutic assistance because of punctual communication, which reduces time and costs. Moreover, TR is performed in the home environment, where individuals are relaxed and confident in performing the proposed exercises [11].

Gandolfi et al. (2016) showed a significant improvement in balance after virtual reality training at home. A systematic review on telehealth for PD concluded that TR effectively improved motor impairment [11]. One study indicated that most interviewed individuals with PD (76%) were willing to participate in a future TR session [18].

The use of TR may increase in the next years due to the appreciation and acceptance of individuals with PD [19]. To the best of our knowledge, two reviews were conducted after the pandemic, in 2022 [20] and 2021 [21]. In one of these reviews [20], the authors included studies involving mobile devices without focusing on TR. In the other review [21], no meta-analysis was performed, and the non-motor symptoms analyzed included those not related to physiotherapeutic rehabilitation (e.g., dysphagia and speech disorders).

The objective of the present study is to evaluate the effects of physical therapy interventions administered remotely in individuals with PD.

## Materials and methods

This systematic review protocol was conducted according to the Preferred Reporting Items for Systematic Review and Meta Analysis Protocols [22]. This systematic review protocol was registered on the Open Science Framework (https://doi.org/10.17605/OSF.IO/ZVPX5-https://osf.io/zvpx5).

### Participants

Studies involving adult individuals (≥ 18 years) diagnosed with Parkinson’s disease (PD), at any stage of the disease, will be included. Studies conducted with participants presenting other associated neurological disorders will be excluded unless data specific to individuals with PD are reported separately.

### Interventions

Studies investigating physical therapy delivered via synchronous or asynchronous telerehabilitation (TR) will be included. Physical therapy interventions using TR either as a standalone approach or in combination with other interventions will also be considered.

### Comparators

Comparators will include conventional face-to-face therapy (physical therapy or other therapeutic approaches), remote interventions not involving physical therapy, or no intervention.

### Outcomes

Studies reporting outcomes related to gait, balance, motor function, functional mobility, or quality of life will be considered for inclusion.

### Types of outcome measures

#### Primary outcomes.

The primary outcomes will be motor function (Unified Parkinson’s Disease Rating Scale [UPDRS]), gait (Freezing of Gait Questionnaire [FOG-Q], gait speed, and stride length, and balance (Push test, Berg balance test, and Timed Up and Go [TUG]).

#### Secondary outcomes.

Secondary outcomes will be the number of falls, considering the time windows of primary studies; the amount and description of adverse effects (e.g., pain, falls, hospitalization, and all causes of death); quality of life (PD Questionnaire-39); and fear of falling.

Results will be considered at specific evaluation moments: baseline (last measure before intervention); post-intervention (first measure after the end of intervention); and follow-up (second measure after the end of intervention).

### Searching methods

#### Electronic searches.

A search strategy was created based on the PRISMA extension for searches (PRISMA-S) [23]. The strategy was developed in the MEDLINE database using free terms and terms from the Medical Subject Headings related to PD, telehealth, rehabilitation, and physical therapy. These terms were adapted to the following databases and trials registrations: MEDLINE (PubMed), EMBASE, Cochrane Central Register of Controlled Trials (Cochrane Register of Studies Online [CENTRAL]), Physiotherapy Evidence Database (PEDro).

All databases and trial registrations will be searched from inception to date without language or publication type restriction. Texts will be translated to English if needed.

The abstracts of conferences and published and unpublished studies from gray literature (i.e., theses and dissertations) will also be verified for additional references. Trial registrations will be searched on ClinicalTrials.gov (www.clinicaltrials.gov).

### Data collection and analysis

#### Study selection.

Two researchers (LC and LS) will independently examine the titles and abstracts of studies and classify as retrieved (eligible, potentially eligible, or unclear) or not retrieved. We will retrieve the full texts of all potentially eligible studies, and two researchers (LV and RT) will independently screen for inclusion and record reasons for excluding ineligible studies. Disagreements will be resolved by consensus or consultation with a third researcher (LM or LP). Duplicates and groups of multiple reports from the same study will be identified and excluded. The selection process will be recorded in detail to complete the PRISMA flow diagram and the table of characteristics of excluded studies [22].

#### Data extraction and management.

A data collection form applied to at least one included study will collect the study characteristics and results. Researchers (LV and RT) will extract the following characteristics of included studies: methods (study design, total duration of study, number of study centers and location, study location, withdraws, and study date); participants (number of participants, mean age, age group, sex, severity of the condition [Hoehn and Yahr Scale], diagnostic criteria, inclusion criteria, and exclusion criteria); interventions (characteristics of intervention and comparisons); results (primary and secondary results specified and collected and time windows); and notes (funding and conflicts of interest).

Two researchers (LV and RT) will independently extract data from the results of included studies. One researcher (LM) will transfer data to the review manager file [24], and a second verification will validate whether data were entered correctly by comparing data from the systematic review with study reports. A second researcher (LP) will also compare the characteristics of studies with those in the study report.

#### Meta-analysis.

Primary and secondary outcomes will be combined, when available, to compare TR (either alone or in combination with another intervention) against a control group (in-person intervention, non-physiotherapeutic remote intervention, or no intervention). Data from post-intervention and follow-up endpoints will also be utilized when available. Meta-analyses will be conducted using a random-effects model, while a narrative synthesis will be performed if meta-analysis is not feasible. The meta-analyses will evaluate the effects of TR compared to control interventions on motor function, gait ability (including speed and stability), postural balance, freezing of gait, fall risk, and quality of life.

Data will be synthesized using the Review Manager 5.4 software. The weighted mean difference will be calculated for continuous outcomes measured with consistent instruments and units. When instruments and units vary, the standardized mean difference (SMD) will be used. For dichotomous outcomes, treatment effects will be assessed using odds ratios, and 95% confidence intervals (95%CI) will be computed for all estimates. Heterogeneity will be assessed by identifying differences between the populations included in the studies after the meta-analysis. Statistical heterogeneity will be assessed using the I² statistic, interpreted as none (0% to 40%), moderate (30% to 60%), substantial (50% to 90%), or considerable (≥75% to 100%).

#### Measures of treatment effect.

If data from rating scales are combined in a meta-analysis, we will insert it with a consistent direction of effect.

#### Risk of bias assessment in included studies.

Two researchers (LM and LP) will independently assess the risk of bias in studies using the Cochrane Handbook for Systematic Reviews of Interventions and Cochrane Risk of Bias tool [25]. Any disagreements will be resolved by consensus or involving another researcher (TR). The risk of bias will be assessed according to the following domains: randomization process; deviations from intended interventions; missing outcome data; measurement of the outcome; selection of the reported result.

#### Assessment of bias in the systematic review.

Potential sources of bias will be judged in this study and summarized in a table of risk of bias. The systematic review will follow this protocol, and any deviations will be justified in the section corresponding to differences between protocol and review.

#### Assessment using GRADE (Grading of recommendations assessment, development, and evaluation).

The GRADE [26] will assess the quality of the evidence to reinforce the importance and impact of methodological bias in the synthesis of results of the systematic review.

#### Dealing with lost data.

Study researchers or sponsors will be contacted to verify the main characteristics of the study (e.g., when a study is identified as abstract only). In the GRADE classification, we will also consider whether the contact was impossible and missing data introduced serious bias.

#### Subgroup analysis.

If the number of included studies is sufficient, the following variables: age (< 70 years and ≥ 70 years); stage of disability (Hoehn and Yahr scores 1–3, 2–3, or 2–4); and dose of therapy (< 2000 and > 2000 minutes of therapy).

#### Sensitivity analysis.

Sensitivity analyses will be performed if any missing data are suspected of bias and to assess heterogeneity caused by outlying studies. In addition, sensitivity analyses excluding studies with or a high risk of bias regarding allocation concealment.

Although the primary analyses will follow the pre-specified protocol, certain aspects of the methodology — such as handling of missing data — may require adjustment based on data characteristics encountered during the study. Any such modifications will be transparently documented and justified. Furthermore, if exploratory analyses are conducted beyond the primary hypotheses, these will be explicitly labeled as such to distinguish them from confirmatory tests.

#### Article summary.

**Fig 1:**
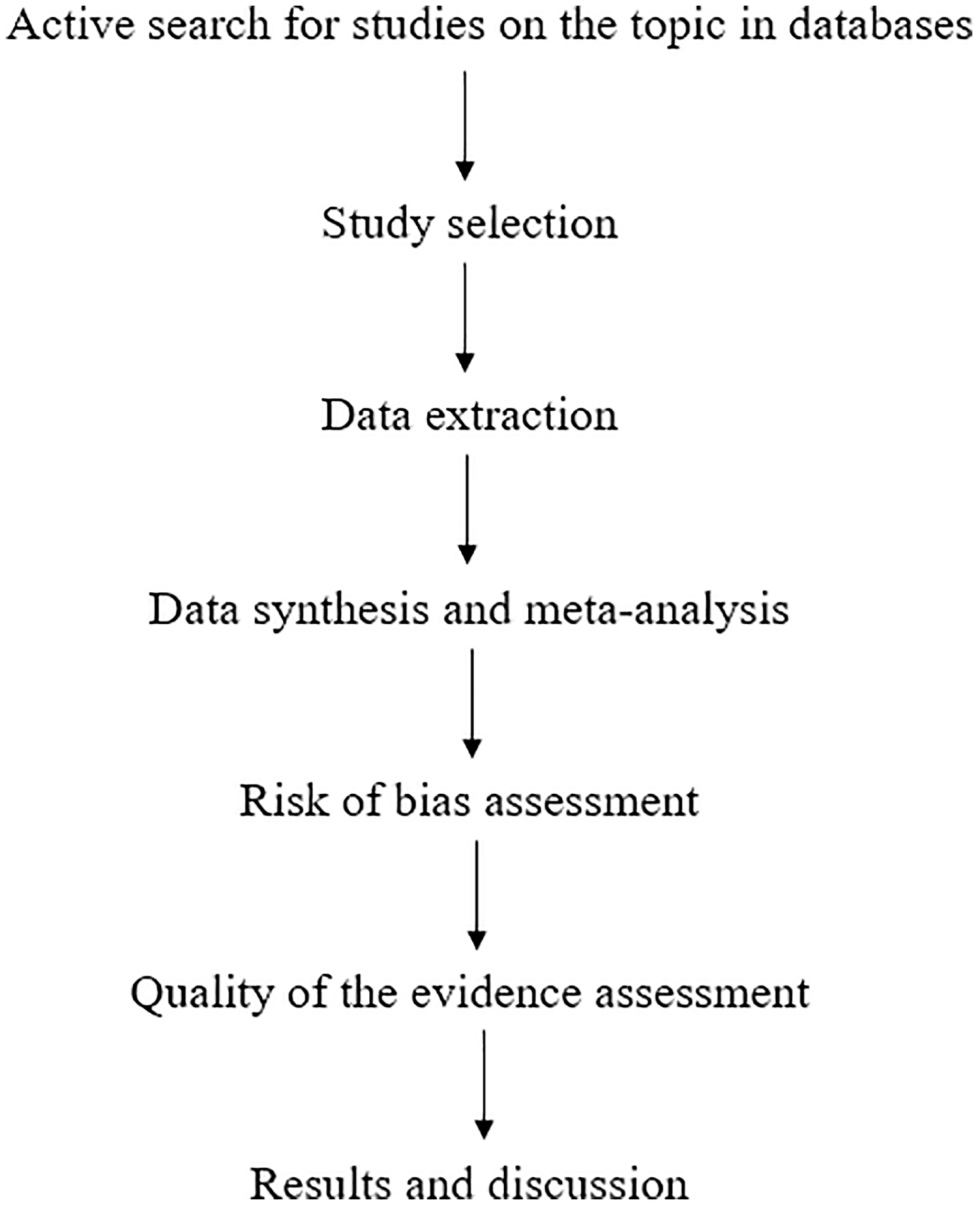
Flowchart of the study to be conducted by PRISMA.

TR may be an alternative for delivering rehabilitation because it excludes travel time, minimizes financial expenses, and promotes a comfortable treatment at home. Due to the COVID-19 pandemic, digital technologies and strategies (including TR) expanded to improve the response of the health system [10,26,27]. According to the 2024 Telerehabilitation Guidelines, the benefits of TR include: improved adherence to treatment and completion of prescribed tasks (e.g., home exercise, mobilization); greater flexibility in care models (e.g., enhanced patient and clinician choice of delivery methods such as videoconferencing, store-and-forward, hybrid); and increased patient satisfaction. In this context, the present study is significant for individuals with PD, as it aims to enhance treatment and management of the disease in the home setting, whether synchronous or asynchronous. This study will enable an evaluation of the applicability of TR to both motor and non-motor outcomes, and identify which outcomes show greater efficacy. Additionally, it will provide insights into the facilitators and barriers associated with this treatment [28]. Therefore, Fig 1 illustrates the flowchart of the study developed in accordance with the PRISMA guidelines.

#### Strenghts and limitations.

- *Strenghts:*

This study will include research involving physical therapy conducted through either synchronous or asynchronous TR, as well as studies involving individuals with PD who have undergone TR.

Outcomes will be assessed at baseline, post-intervention, and during follow-up periods. The analysis will encompass both motor outcomes—such as motor function, gait, and balance—and non-motor outcomes—such as adherence, quality of life, and fear of falling.

The most widely used medical databases will be searched for relevant studies. The study aims to provide specific and meta-analyzed data on physiotherapeutic TR for PD.

- *Limitations:*

Given that our study will encompass all individuals with PD who have undergone TR, there is a potential for bias related to variations in age and disease staging. However, it is important to emphasize that our objective is to assess the overall effect of TR on PD rather than focusing on specific patient subsets. Additionally, we will categorize the results into subgroups to mitigate this potential bias.

Another limitation is the lack of differentiation among TR modalities (i.e., image-based, sensor-based, or virtual reality). We will aggregate TR as a unified category, including studies that have utilized any form of TR.

#### Ethics and dissemination.

The results of the systematic review will be disseminated via publication in a peer-reviewed journal and presented at a relevant conference. The data we will use do not include individual patient data, so ethical approval is not required.

## Patient and public involvement

No patient involved. As this is a systematic review, patients are only involved in studies previously carried out, which will be recruited according to the eligibility criteria.

## Registration details

This systematic review protocol was registered on the Open Science Framework (https://doi.org/10.17605/OSF.IO/ZVPX5-https://osf.io/zvpx5).

## Supporting information

S1 File Search strategy. (DOCX)
