# Supplementary material for: Effects of telerehabilitation-based physical therapy for individuals with Parkinson’s disease: A systematic review protocol
Source: PLoS One. 2026 Mar 6;21(3):e0342771. doi: 10.1371/journal.pone.0342771 (PMC12965527; doi:10.1371/journal.pone.0342771)
Supplement: S1 File — (DOCX) [file pone.0342771.s001.docx]

**SEARCH STRATEGY**

The search strategy will include the following terms:

Condition Descriptors: parkinson, parkinson’s, parkinson disease, parkinson’s disease, parkinsons, PD, Wolff-Parkinson-White Syndrome, Lewy Body Disease, autosomal dominant juvenile parkinson disease, autosomal recessive juvenile parkinson disease, experimental Parkinson disease, experimental Parkinson diseases, diseases, experimental parkinson.

Intervention Descriptors: telerehabilitation, telerehabilitation physical therapy, telereabilitation, telehealth, telehealth physical therapy (no results in Cochrane), telemedicine telehealth, remote intervention, telerehabilitations, tele-rehabilitation, tele rehabilitation, tele-rehabilitations, remote rehabilitation, rehabilitation, remote, rehabilitations, remote, remote rehabilitations, rehabilitation, virtual, rehabilitations, virtual, virtual rehabilitations, mobile health, health, mobile, mHealth, telehealth, eHealth.

Study Type Descriptors: trial, clinical trial, control trial, control clinical trial, random control trial, random trial, random clinical trial, random sample, random, randomized controlled trial, controlled clinical trial, experimental, experimental design, experimental study, experimental research, placebo, groups, quasi-experimental, quasi-experimental study, quasi-experimental design, quasi-experimental research, quasi experimental studies, cross over, cross over design, cross over study, cross over trial, cross over clinical trial.

Search string: parkinson OR parkinson’s OR parkinson disease OR parkinson’s disease OR parkinsons OR PD OR Wolff-Parkinson-White Syndrome OR Lewy Body Disease OR autosomal dominant juvenile parkinson disease OR autosomal recessive juvenile parkinson disease OR experimental Parkinson disease OR experimental Parkinson diseases OR diseases, experimental parkinson
AND telerehabilitation OR telerehabilitation physical therapy OR telereabilitation OR telehealth OR telehealth physical therapy OR telemedicine telehealth OR remote intervention OR telerehabilitations OR tele-rehabilitation OR tele rehabilitation OR tele-rehabilitations OR remote rehabilitation OR rehabilitation, remote OR rehabilitations, remote OR remote rehabilitations OR rehabilitation, virtual OR rehabilitations, virtual OR virtual rehabilitations OR mobile health OR health, mobile OR mHealth OR telehealth OR eHealth
AND trial OR clinical trial OR control trial OR control clinical trial OR random control trial OR random trial OR random clinical trial OR random sample OR random OR randomized controlled trial OR controlled clinical trial OR experimental OR experimental design OR experimental study OR experimental research OR placebo OR groups OR quasi-experimental OR quasi-experimental study OR quasi-experimental design OR quasi-experimental research OR quasi experimental studies OR cross over OR cross over design OR cross over study OR cross over trial OR cross over clinical trial.
